# Primary thymic carcinoma with adenoid cystic carcinoma-like features: A case report and literature review

**DOI:** 10.1097/MD.0000000000021531

**Published:** 2020-07-31

**Authors:** Mai-Qing Yang, Lin-Lin Bai, Zhao Wang, Wen-Jing Huang, Gui-Yang Jiang, Hong-Tao Xu

**Affiliations:** a Department of Pathology, The First Hospital and College of Basic Medical Sciences, China Medical University, Shenyang; b Department of Pathology, Changyi People's Hospital, Changyi; c Department of Pathology, Shenyang 242 Hospital, Shenyang; d Department of Pathology, General Hospital of Heilongjiang Land Reclamation Bureau, Harbin, China.

**Keywords:** adenoid cystic carcinoma-like features, mediastinum, thymic tumor, thymoma

## Abstract

**Rationale:**

Thymic carcinoma with adenoid cystic carcinoma-like features is a special subtype of thymic adenocarcinoma, and the occurrence of this condition is extremely rare. Herein, we report a case of primary thymic carcinoma with adenoid cystic carcinoma-like features in a young man.

**Patient concerns:**

A 38-year-old man had an incidental finding of space-occupying lesion in the anterior mediastinum during a routine health examination. The patient complained of occasional mild chest tightness during hot weather but had no obvious cough, sputum, chest pain, or fever. Contrast-enhanced computed tomography scan of the chest revealed a space-occupying lesion in the anterior mediastinum, which is likely benign.

**Diagnosis:**

The lesion was diagnosed as a primary thymic carcinoma with adenoid cystic carcinoma-like features.

**Intervention:**

The patient underwent thoracoscopic resection of left anterior mediastinal mass and enlarged resection of thymectomy and mediastinal fat in our hospital.

**Outcomes:**

The postoperative course was uneventful.

**Lessons:**

The tissue characteristic of this tumor was extremely similar to that of adenoid cystic carcinoma. A precise pathological examination is extremely important to prevent misdiagnoses of the lesion as adenoid cystic carcinoma or other thymic tumors. Immunohistochemical staining is extremely useful for the pathological and differential diagnoses of this tumor.

## 1 Introduction

Thymic carcinomas are rare malignant thymic epithelial tumors with several histologic subtypes. Thymic adenocarcinoma is a rarer heterogeneous subtype of malignant thymic carcinoma. Patients with thymic adenocarcinoma have a median age of 52.6 years, and the male-to-female ratio is 2:1. In the International Thymic Malignancy Interest Group database, thymic adenocarcinomas only account for 0.48% (29 of 6097) of all thymic epithelial neoplasm cases.^[1]^ Thymic carcinoma with adenoid cystic carcinoma-like features (TCACC) is an extremely rare subtype of thymic adenocarcinoma, and this condition was first reported by Di Tommaso et al in 2007.^[2]^ The histological characteristics of TCACC are extremely similar to those of adenoid cystic carcinoma of the salivary gland. To the best of our knowledge, only 7 cases of this type of tumor had been reported previously.^[2–5]^ The etiology and clinicopathological factors of this tumor are not clearly elucidated. Herein, we report a case of TCACC in a 38-year-old male patient. Moreover, the features of TCACC were assessed and summarized.

## 2 Case presentation

### 2.1 Ethic approval

This study was approved by the institutional review board of China Medical University for human studies. The ethical board approval number is LS[2018]016. A written informed consent was obtained from the patient for the publication of this case report and accompanying images.

### 2.2 Clinical history

**Figure 1:**
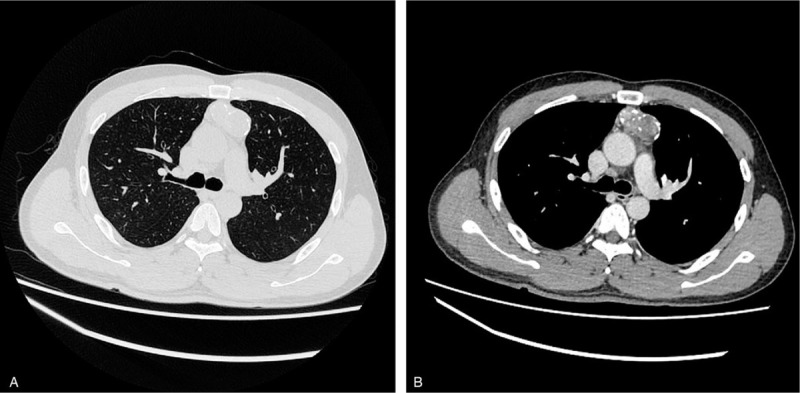
Computed tomography scan of the chest. Computed tomography scan revealed a single, lobulated, well circumscribed, round mass, with an uneven density in front of the pulmonary conus in the anterior mediastinum. The mass was approximately 4.0 cm × 2.2 cm in size, with a smooth outline and multiple calcifications at the edge.

A 38-year-old man was admitted to our hospital for further treatment due to an incidental diagnosis of a space-occupying lesion in the anterior mediastinum in Aug 2019. The patient did not receive any systematic treatment. Moreover, he had a smoking history of 20 cigarettes per day for 10 years but no tumor or surgical history. The patient experienced occasional mild chest tightness during hot weather but had no obvious cough, sputum, chest pain, or fever. Routine laboratory examination, physical examination, and pulmonary function test had no positive findings. Contrast-enhanced computed tomography (CT) scan revealed a single, lobulated, well-circumscribed round mass, with an uneven density in front of the pulmonary conus in the anterior mediastinum. The tumor was approximately 4.0 cm × 2.2 cm in size, with a smooth outline and multiple calcifications at the edge (Fig. 1). The plain CT scan values were about 18 to 33 and 27 to 92 HU after enhancement. The heart was normal in size. The patient did not present with any abnormalities in the soft tissues of the chest wall, and enlargement of the lymph nodes was not observed. The tumor was considered benign based on clinical and imaging examinations and was then resected via thoracoscopy. During surgery, tumor samples were obtained for the preparation of frozen sections for pathological evaluation. Using the intraoperative frozen sections, the tumor was diagnosed as low-grade malignant tumor, which needed routine paraffin section staining and immunohistochemical identification and classification after operation. Then, further resection of the enlarged thymectomy and mediastinal fat was performed. The patient did not receive any postoperative adjuvant therapies.

### 2.3 Immunohistochemical staining

The resected tumor tissue was fixed with 10% neutral-buffered formalin, embedded into paraffin blocks, and cut into 4-μm sections. The tissue sections were stained with hematoxylin and eosin for histological evaluation. Then, some sections were immunostained with ready-to-use primary antibodies against CD117, CD5, CD20, broad-spectrum cytokeratin (CK), CK7, PAX8, P63, actin (SM), chromogranin A, CD56, S-100, synaptophysin, thyroid transcription factor-1 (TTF-1), vimentin, thyroglobulin, terminal deoxynucleotidyl transferase (TdT), and Ki-67. These antibodies were purchased from Maixin, Fuzhou, China. After incubation with the primary antibody, the presence of antibodies was assessed using the streptavidin–peroxidase method. Positive and negative controls were used accordingly to prevent false positivity and negativity. Tumor sections were also stained with Alcian blue-periodic acid Schiff (AB-PAS).

### 2.4 Morphological and immunohistochemical findings

Grossly, the specimen comprised a round, well-defined nodular lesion that is 5.0 cm × 3.5 cm × 2.0 cm in size. The capsule was complete and intact. Most tissues were soft, and some focal areas were calcified and hard, with an eggshell-like outline, containing fish-like tissues. The cut section showed multi-cysts with a mixture of fibrotic yellow tissues. The tumor mass was circumscribed, and the resection margins were tumor free.

**Figure 2:**
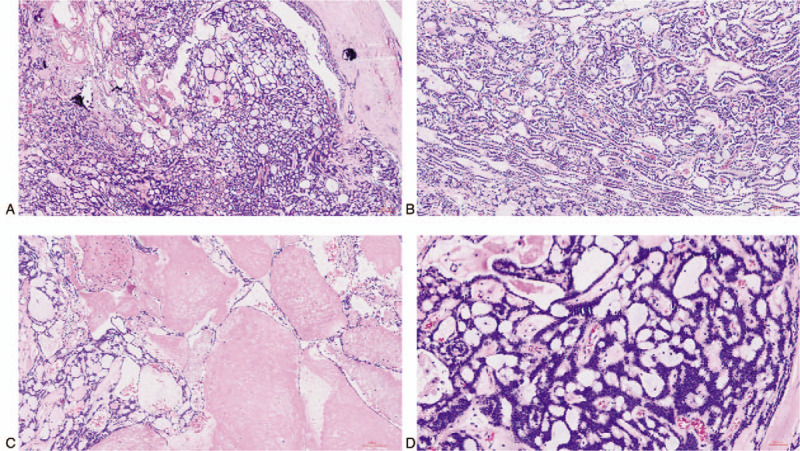
Histological features of the primary thymic carcinoma with adenoid cystic carcinoma-like features. (A) The tumor was well circumscribed and enclosed by thick fiber tissues with hyaline degeneration and calcification at some regions. Moreover, it had multi-cysts or cribriform structures, and some structures were filled with mucoid substance or homogenous pink secretion (hematoxylin and eosin [H&E] staining, 40×). (B) Some tumor regions had a papillary and solid cord-like structure with homogenous pink basement membrane material between the cell cords (H&E staining, 100×). (C) Some cysts were dilated and filled with homogenous pink secretion (H&E staining, 100×). (D) The tumor had uniform-appearing basaloid cells with bland oval nuclei and well-arranged pseudocysts without glandular epithelial differentiation (H&E staining, 200×).

Microscopically, the tumor was well circumscribed and enclosed by thick fiber tissues with hyaline degeneration and calcification at some regions. The tumor had multi-cysts or cribriform structures, which strongly resembled that of an adenoid cystic carcinoma of the salivary gland. Some cysts were dilated. The cysts or cribriform structures were filled with mucoid substance or homogenous pink basement membrane material. Some tumor regions had a small amount of papillary and solid cord-like structures with homogenous pink basement membrane material between the cell cords. The tumor had uniform-appearing basaloid cells with bland oval nuclei and well-arranged pseudocysts. Unlike adenoid cystic carcinoma, which has 2 types of tumor cells with glandular epithelial and myoepithelial differentiation, the tumor cells in the present case were uniform and formed pseudocysts without obvious glandular epithelial differentiation. The mitotic figure in the whole tumor was rare (Fig. 2). No tumor cell involvement was observed in the resected lymph nodes.

**Figure 3:**
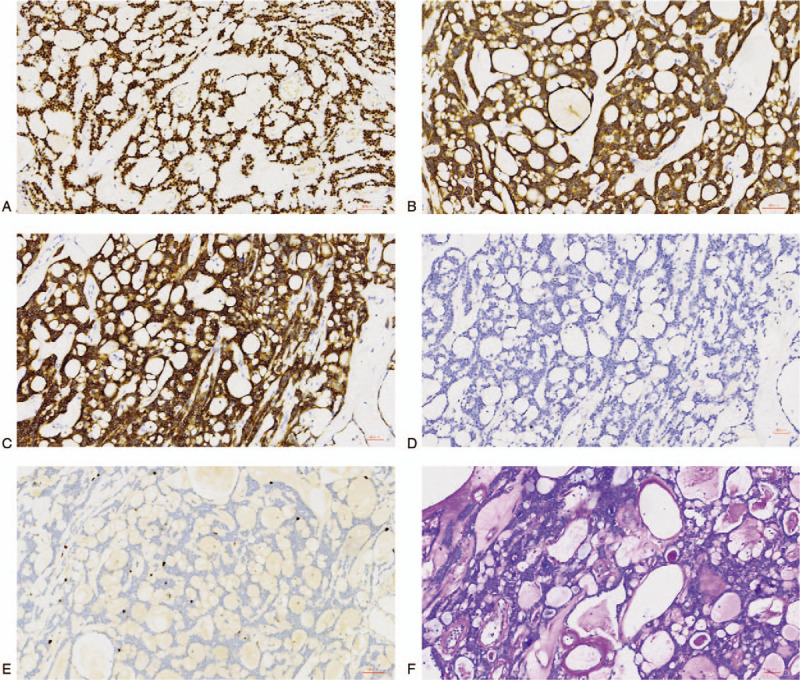
Immunohistochemical and Alcian blue-periodic acid Schiff (AB-PAS) staining of thymic carcinoma with adenoid cystic carcinoma-like features. (A–C) Immunohistochemical staining showed positivity for P63 (A), CK7 (B), and CD117 (C) in most tumor cells (200×). (D) Immunohistochemical staining revealed negativity for synaptophysin in the tumor cells (200×). (E) The Ki-67 index of the tumor cells was less than 10% (200×). (F) The mucoid substance and homogenous pink basement membrane material were positive for AB-PAS staining (200×).

Immunohistochemically, all the neoplastic cells were strongly positive for CK, CK7, P63, and CD117, which indicated a thymic epithelial origin and uniform cell type. In addition, the tumor cells were multifocally moderately positive for CD56, scattered moderately positive for S-100, and negative for CD20, PAX8, actin (SM), chromogranin A, synaptophysin, TTF-1, vimentin, and thyroglobulin. The Ki-67 index of all tumor cells was less than 10%. No thymocytes were detected because immunostaining for TdT was negative. The mucoid substance and homogenous pink basement membrane material in the cysts or between the cell cords were positive for AB-PAS staining (Fig. 3).

## 3 Discussion

Based on the clinical information, histological features, and immunohistochemical staining results described above, the final diagnosis was TCACC.

Thymic carcinomas are rare thymic epithelial malignant tumors with several histologic subtypes.^[6]^ TCACC is a subtype of thymic adenocarcinoma, and the occurrence of this condition is extremely rare. In 2007, Di Tommaso et al have first reported 4 cases of TCACCs.^[2]^ To the best of our knowledge, only 8 cases of TCACC, including the present case, were reported. The clinicopathological features of these cases are summarized in Table 1. Of the patients with TCACCs, 6 were men and 2 were women aged 38 to 77 (mean age: 61) years. The patient in the present case is the youngest among all the patients. Two male patients had a long history of smoking. The diameters of the tumor ranged from 2 to 14 (mean: 9) cm. Only one patient presented with distant metastasis.^[4]^

**Table 1:**
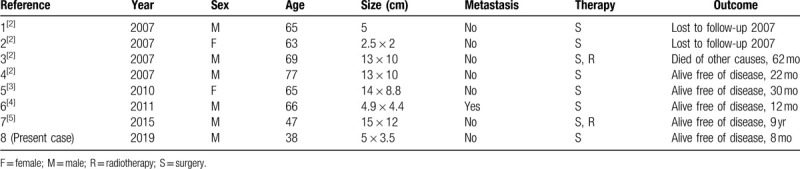
Summary of primary thymic tumors with adenoid cystic carcinoma-like features.

The histological features of TCACC are extremely similar to those of adenoid cystic carcinoma of the salivary gland. TCACC comprised nests of basaloid cells with varying numbers of pseudocysts filled with homogeneous basement membrane material. Slight to moderate nuclear atypia was observed. Tumor necrosis or perineural invasion is not a prominent feature.^[7]^ The morphological features and biological behavior both indicate that TCACC was a low-grade thymic tumor. TCACC must be distinguished from other primary or metastatic tumors with similar histological features, such as adenoid cystic carcinoma, type A thymoma, and thyroid papillary carcinoma. The tumor that should be distinguished from TCACC is metastatic adenoid cystic carcinoma because of its extremely similar cribriform tissue structure. Detailed medical history taking and clinical examinations are important in ruling out metastatic adenoid cystic carcinoma. Morphologically, unlike the uniform basaloid tumor cells of TCACC, adenoid cystic carcinoma has 2 types of tumor cells with glandular epithelial and myoepithelial differentiation. In addition, the translocation of the *MYB* gene is considered a characteristic of adenoid cystic carcinoma and is extremely useful in differential diagnosis.^[8]^ Adenoid cystic carcinoma is a common tumor of the salivary glands of head and neck. However, it is rarely observed in other organs, such as the breast, trachea, lung, prostate, and Bartholin's gland.^[3,9–13]^ However, no study has reported about primary adenoid cystic carcinoma in the thymus to date. Type A thymoma may also comprise adenoid or microcyst components. But it is often positive for PAX8 and CD20, but negative for CD117, which is helpful in distinguishing such condition from TCACC. Meanwhile, TCACC is negative for neuroendocrine markers, such as chromogranin A and synaptophysin, and this immunohistochemical feature is useful in ruling out neuroendocrine tumors. In the present case, TCACC also comprised a small amount of papillary and solid cord-like structures, which must be differentiated with metastatic thyroid papillary cancer. Negativity for TTF-1 and thyroglobulin ruled out the possibility that the tumor is of thyroid origin.

Surgical resection of TCACC is appropriate and feasible. Six of eight patients with TCACC underwent surgery alone, and 2 patients were treated with combined therapy (surgery and radiation). The definite histopathologic prognostic factors of this tumor have not been fully elucidated due to its rarity. In the 8 cases of TCACC, including the current case, only 1 patient died of myeloid leukemia 5 years after, and 2 patients were lost to follow-up. The patient with the longest follow-up is alive and free of the disease for 9 years after diagnosis.^[2–5]^ In the present case, the patient underwent surgery alone and is alive and free of the disease for 8 months after diagnosis.

To sum up, herein, we report a case of TCACC in a young man. This type of tumor is rare and noteworthy of attention from surgeons and pathologists. Precise pathological examination and immunohistochemical staining are useful for the accurate diagnosis of this tumor.

## Acknowledgments

We would like to thank Editage (www.editage.cn) for English language editing.

## Author contributions

**Study design:** Hong-Tao Xu.

**Methodology:** Mai-Qing Yang, Lin-Lin Bai, Zhao Wang, Wen-Jing Huang, Gui-Yang Jiang.

**Writing – original draft:** Mai-Qing Yang, Hong-Tao Xu.

**Writing – review & editing:** Hong-Tao Xu, Gui-Yang Jiang.
